# Improving the Sensitivity and Functionality of Mobile Webcam-Based Fluorescence Detectors for Point-of-Care Diagnostics in Global Health

**DOI:** 10.3390/diagnostics6020019

**Published:** 2016-05-17

**Authors:** Reuven Rasooly, Hugh Alan Bruck, Joshua Balsam, Ben Prickril, Miguel Ossandon, Avraham Rasooly

**Affiliations:** 1 Western Regional Research Center, Agricultural Research Service, U.S. Department of Agriculture, Albany, CA 94706, USA; reuven.rasooly@ars.usda.gov; 2 Department of Mechanical Engineering, University of Maryland College Park (UMCP), College Park, MD 20742, USA; bruck@umd.edu; 3 Division of Chemistry and Toxicology Devices, Office of In Vitro Diagnostics and Radiological Health, FDA, Silver Spring, MD 20993, USA; joshua.balsam@fda.hhs.gov; 4 National Cancer Institute, Rockville, MD 208503, USA; ben.prickril@nih.gov (B.P.); ossandom@mail.nih.gov (M.O.)

**Keywords:** webcams, CCD cameras, mobile phones, fluorescence imaging, flow cytometry, rare cells, resource-poor settings, image enhancement, background subtraction, pixel binning, global health

## Abstract

Resource-poor countries and regions require effective, low-cost diagnostic devices for accurate identification and diagnosis of health conditions. Optical detection technologies used for many types of biological and clinical analysis can play a significant role in addressing this need, but must be sufficiently affordable and portable for use in global health settings. Most current clinical optical imaging technologies are accurate and sensitive, but also expensive and difficult to adapt for use in these settings. These challenges can be mitigated by taking advantage of affordable consumer electronics mobile devices such as webcams, mobile phones, charge-coupled device (CCD) cameras, lasers, and LEDs. Low-cost, portable multi-wavelength fluorescence plate readers have been developed for many applications including detection of microbial toxins such as *C. Botulinum* A neurotoxin, Shiga toxin, and *S. aureus* enterotoxin B (SEB), and flow cytometry has been used to detect very low cell concentrations. However, the relatively low sensitivities of these devices limit their clinical utility. We have developed several approaches to improve their sensitivity presented here for webcam based fluorescence detectors, including (1) image stacking to improve signal-to-noise ratios; (2) lasers to enable fluorescence excitation for flow cytometry; and (3) streak imaging to capture the trajectory of a single cell, enabling imaging sensors with high noise levels to detect rare cell events. These approaches can also help to overcome some of the limitations of other low-cost optical detection technologies such as CCD or phone-based detectors (like high noise levels or low sensitivities), and provide for their use in low-cost medical diagnostics in resource-poor settings.

## 1. Introduction

Over 80% of the world population lives in low and middle income countries (LMICs) [1]. In recent years healthcare interventions in LMICs have focused on infectious diseases such as acute lower respiratory infections, HIV/AIDS, Diarrheal Diseases, tuberculosis, or malaria. The development of low-cost detection/diagnostic technologies for global health has, not surprisingly, maintained this focus [2]. However, noncommunicable diseases (NCDs) present a larger disease burden in these countries. These include heart disease, stroke, cancer, chronic respiratory diseases, and diabetes. Collectively these are the leading cause of death and disability in LMICs, but attract less than 2 percent of global health aid [3]. Unlike many infectious diseases where the detection of pathogens is relatively simple, detection and diagnosis of many NCDs is more challenging and complex, and early disease detection and diagnosis is critical for effective care management. Despite the efforts to develop medical detection technologies for NCDs for global heath [4], current detection technologies are often too complex, expensive, and unsustainable to address the needs of LMICs.

Resource-poor countries and regions require effective, low-cost diagnostic devices for accurate identification and diagnosis of health conditions [2]. Essential biomedical technologies for LMICs must be affordable and sustainable in low-resource settings. Therefore, these technologies must be simple, reasonably mobile, and based on components and expertise readily available in these settings. In addition, in order to serve large populations the technologies must be suitable for Point-of-Care Testing (POCT), which is medical diagnostic testing performed on-site.

### 1.1. Mobile Technologies for POCT

In recent years, many mobile POCT devices have been introduced including POCT devices based on low-cost consumer electronics technologies such as smartphones or tablets, which have sophisticated computing capabilities and are widely available in LMICs [5]. These devices have been integrated into several types of transducers to provide new capabilities such as smartphone attachments for stethoscopes [6,7,8], smartphone-based ambulatory blood pressure monitoring [8,9,10,11,12], mobile point-of-care ultrasound [13], or digital microscopy [14,15,16,17,18,19,20,21,22]. New assay technologies such as lateral flow, paper-based microfluidics [23], phone-based colorimetric readers [24], or lab-on-a-chip (LOC) were used for the development of sensitive, low-cost biological assays [25]. These include various chemical [26,27] and biological assays for DNA amplification outside of laboratory environments [28] and analysis of toxin activities and immunological assays for many types of medical settings [29,30].

### 1.2. Optical Detection and Analysis

Many biological diagnostic techniques use optical methods for research, clinical, and industrial analysis, including light absorbance, fluorescence, polarization colorimetry, spectrometry, and luminescence [31]. Several types of optical detectors are used for biodetection. For applications requiring sensitive light measurements, such as plate readers for flow cytometry, avalanche photodiodes [32,33,34,35] and photomultipliers [36,37,38,39,40,41] are used. However, these are point detectors that are spatially limited and therefore very slow when analyzing large sample volumes. More recently, optical detectors with arrays of point detectors, referred to as “pixels,” employ charge-coupled device (CCD), or complementary metal–oxide–semiconductor (CMOS) detectors, have been used to create “images” that enable larger areas to be interrogated very quickly [42,43,44,45]. The main advantage of these “imaging sensors” over photodiodes or photomultipliers (which are only capable of “spot” analysis of samples) is the ability to analyze light from an area large enough to cover the entire surface of a multi-channel device or a lab on a chip (LOC)-based assay/array [46,47,48]. This enables “parallel” multi-sample analysis where large sample volumes can be analyzed simultaneously.

Optical-based detection is especially attractive for mobile POCT due to its simplicity, size, sensitivity, and cost. Many consumer electronics and wireless telecommunications employ optical detectors [49], including smartphones, tablets, webcams, flatbed scanners, and computer gaming consoles. All of these are essentially powerful portable computers with imaging capabilities, and all have the potential to be converted to low-cost, portable optical imaging platforms for mobile POCT. Moreover, many biological assays and clinical tests (e.g., ELISA, microarray analysis, and DNA sequencing) use optical detectors, so there are already many applications, reagents, and assays that can be adapted to POCT for use in resource-poor settings. Examples of such devices include readers for lateral flow immuno-chromatographic assays [50], fluorescence detectors [51,52,53], wide-field fluorescent microscopes [54], lens-free microscopes [55], capillary array for immunodetection [56], and fluorescent imaging cytometers [57]. More recently, a smart phone-based surface plasmon resonance biosensor has been developed [58].

### 1.3. Applications for Mobile Optical Detectors

Numerous biosensing modalities have been applied for mobile devices including various microscopy technologies [19,59,60,61,62,63], fluorescence biosensors [61,64,65,66,67,68,69,70,71], spectroscopy [70,72,73], diffraction [74,75,76,77,78], colorimetric biosensors [79,80,81,82,83,84,85,86,87,88,89,90,91,92,93,94,95,96,97,98,99], chemiluminescence detection [100,101,102,103,104], electrochemiluminescence detection [23,105], SPR biosensors [106,107], electrochemical biosensors [23,108,109,110,111,112,113], and potentiometric biosensors [114,115]. One of the most commonly used immunological assays is the Enzyme-Linked Immunosorbent Assay (ELISA) [116,117], originally designed as an optical detection laboratory test to replace the radioimmunoassay technique. Many portable immunology-based diagnostic devices are based on CCD detection systems combined with LOC [29,43,45,47,118,119,120,121], which have sensitivities comparable to laboratory ELISA plate readers for many applications, including microchip ELISA-based detection of the ovarian cancer HE4 biomarker in urine [122]. A smartphone with a CMOS detector has been used as a portable ELISA detector [76], utilizing the integrated phone camera as a spectrometer for detection of IL-6, a protein used diagnostically for several types of cancer, as well as the Ara h 1 protein, one of the principle peanut allergens. More recently, a portable solar thermal PCR was developed that analyzes fluorescence levels imaged by a smartphone or tablet [123] and was used for the diagnosis of Kaposi’s sarcoma [124].

Mobile optical detectors combined with LOC technologies have also been developed for immunodetection of infectious diseases for POCT, including a smartphone dongle combined with microfluidics [125], a smartphone-based detector used for protein microarray-based fluorescence immunoassay for detection of multiple biomarkers in milk [126], and an ELISA 96-well plate reader [127].

A simple CCD-based detector combined with LOC was developed for immunological detection of microbial toxins, including the detection of staphylococcal enterotoxins utilizing densitometry [128,129]. This system was also automated for high throughput toxin detection [130], including fluorescent detection of toxin activity for botulinum neurotoxin A (BoNT-A) [29,47,48] and analysis of Shiga toxin 2 (Stx2) activity [49]. Such technologies are particularly useful in resource-poor settings [29,30]. In recent years several low-cost portable flow cytometers based on webcams have been described [131,132,133,134].

Flow cytometry is another application using optical detection and LOC technology, where high-powered optics are also necessary to achieve microscopic imaging that enables detection of individual cells. In flow cytometry, cell components are fluorescently labeled, separated from each other in a flow field, and then excited by a laser to emit light from the fluorescence label. There are many clinical applications for flow cytometry, including monitoring HIV infection through CD4 T-cell counting, leukemia and lymphoma phenotyping, detection of minimal residual cancer disease, and diagnosis of immunodeficiency disorders. Conventional flow cytometers are based on hydrodynamic focusing of the cells to a narrow flow for detection by individual photomultipliers. However, one limitation of narrow flow is a low flow rate due to the high hydrodynamic resistance and pressure constraints of the cell. This ultimately limits the device to small volumes or long analysis times, which is not practical for applications such as rare cell detection.

Imaging CCD and CMOS sensors have also been used for optical detection combined with classical flow cytometry [135]. Imaging-based cytometry can also provide multispectral imagery related to the cell morphology [136], protein co-localization on cells [137]. and counting of circulating tumor cells (CTCs) [138]. An optofluidic fluorescence imaging cytometer using a smartphone with a spatial resolution of ~2 μm has also been described [57,61,139]. While very mobile and versatile, the flow rate of this system is low (e.g., ~1 μL/min), which limits analysis to small volumes. In addition, such devices are often limited in their sampling rates (e.g., many smartphones are limited to 30 fps). In addition to smartphone-based cytometers, webcam-based, portable fluorescence imaging flow cytometers for high flow rates (e.g., 10 mL/min) have been developed, which enable higher volumes of cells to be analyzed in order to detect and count rare cells; cell concentrations may be lower than 1 cell/mL [131,140,141].

### 1.4. The Limitations of Low-Cost Mobile POCT Optical Detectors

The fundamental issue in utilizing imaging devices found in consumer electronics for mobile POCT is that the light signals from most biological assays are relatively low, especially for low-level analytes. Thus, high sensitivity detection is required. However, low-cost CMOS and CCD detectors have low sensitivities and high background noise compared to the more expensive imaging technologies or photomultipliers found in laboratory systems. Therefore, in order to be used for POCT, their sensitivity for low-light imaging has to be improved. Additionally, these imaging sensor have limited control and versatility. For example, the lenses on mobile phones are not removable or interchangeable, the aperture of the lens is fixed, and in many low-end devices the number of frames per second (which determine time resolution and image signal-to-noise ratio) is fixed (e.g., 30 fps). Some webcams are more versatile, with removable and exchangeable lenses as well as control of exposure time and framing rate. Several technologies to increase the sensitivity of CCD or CMOS imagers have also been developed, including cooling the CCS/CMOS to reduce thermal noise, the use of image intensifier to increase signal, and the use of electron multiplier CCD (EMCCD) with an electron multiplying (EM) register added to the end of the normal serial register [142]. However, the complexity and high cost of these devices limit their application for POCT, especially in global health and low-resource settings. Alternatively, simpler solutions can be pursued to improve sensitivity such as replacing the native lens of a webcam with a higher aperture lens, increasing the power of the excitation source in fluorescent detection, and using longer exposure times to detect low signal. However, these solutions often increase the noise in the image resulting in the need to develop new imaging approaches to increase the sensitivity of CCD or CMOS imagers.

## 2. Mobile Imaging Fluorescence Detectors

Because of their high sensitivity and simplicity, fluorescence-based assays are ideal for low sensitivity devices. Plate readers are commonly used with optical detectors for immunodetection (e.g., ELISA) or chemical assays for multi-analyte detection (e.g., 96-well plates). Several optical modalities are used in plate readers including absorbance, fluorescence, or luminescence detected using sensitive optical detectors (e.g., photomultipliers).

### 2.1. Basic Configuration of Mobile Imaging Fluorescence Detectors

**Figure 1:**
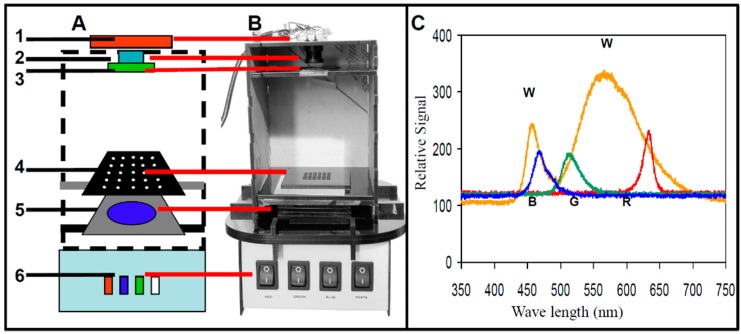
Webcam-based fluorescence plate reader. (**A**) A schematic configuration of the webcam-based plate reader with the main system components highlighted in the schematic: (1) a webcam camera mounted in an acrylic box, (2) interchangeable lens with (3) a green band-pass emission filter mounted on the end of the lens, (4) black acrylic sample chip, (5) blue band-pass excitation filter, and (6) multiwavelength LED. (**B**) Photo of webcam-based plate reader. (**C**) Spectrometric excitation spectra of the multi-wavelength LED for the white (W), blue (B), green (G), and red (R) LED illumination.

The configuration of a simple mobile imaging fluorescence plate reader [46,47,48,49,120,143,144,145,146,147,148,149] is shown schematically in Figure 1A. The main components of the optical detection platform are: (1) mobile imaging device (phone, webcam, or camera); (2) lens; (3) emission filter mounted on the end of the lens; (4) assay plate; (5) excitation filter; and (6) multi-wavelength LED. An image of such detector is shown in Figure 1B. In this simple configuration, the excitation source is directly in line with the detector, so good quality excitation and emission filters are essential for blocking excitation light to keep it from reaching the detector, while still allowing the fluorescence emission to be captured.

### 2.2. Optical Detectors

To increase sensitivity in this setup, a sensitive detector such as Point Grey Research *Chameleon* camera equipped with a C-mount CCTV lens (Pentax 12 mm f/1.2) was used as the photodetector [49]. The advantage of using this camera as a detector is its high sensitivity and range of exposure times. In typical low-cost webcams, the exposure time is determined automatically based on the light exposure of the CMOS to adjust the image to a standard video rate of 30 fps, and therefore it cannot be controlled by the user. In addition, the typical lenses of many low-cost webcams have fixed apertures and focal lengths with extended depth of field. In the *Chameleon* camera the lenses are interchangeable, allowing use of lenses with wider apertures and various focal lengths. However, several types of low-cost generic webcams have also been used [128,143,147,150,151]; in addition, for higher sensitivity detection, more expensive cooled CCD cameras with low dark noise have been used with this configuration [29,30,46,47,48,118,119,120,149,152,153]. Therefore, this simple design is very versatile and can be used for a broad range of fluorescence detectors for a variety of applications.

### 2.3. LED Illumination Module

For optical detection, illumination is a critical component. A versatile illuminator has been described in previous work [48], consisting of a multi-wavelength LED illumination box equipped with white and RGB LEDs that can illuminate in red, green, blue, or white spectral regions (Figure 1C), enabling excitation of multiple fluorophores in a wide excitation range of 450–650 nm (red: 610–650 nm, green: 512–550 nm, and blue: 450–540 nm). This broad wavelength enables the use of many fluorophores used in many different imaging applications [30,48,49,130,143,145,147,148,149,151], including fluorescent microscope illumination [128]. The excitation and emission filters are critical for image quality. For example, for excitation in the blue range, a 20 nm bandpass filter with 486 nm center wavelength was used (D486/20X), and for emission in the green range, a 50 nm bandpass filter with a center wavelength of 535 nm was used (HQ535/50M, both filters from Chroma Technology Corp., Rockingham, VT).

### 2.4. Assay Plate

A laminated assay plate for small volumes (~30 μL) was fabricated as described in our previous work [47,120], where a rigid polymer is used for the core (e.g., 3.2 mm black poly(methyl methacrylate) (PMMA)) and the wells were laser-micromachined. The bottom of the sample wells were laminated with a thin polymer (e.g., polycarbonate (PC) film) bonded with double side adhesive. The thick core provides rigidity to the plate, and the black color minimizes optical reflection and crosstalk between the wells.

### 2.5. Fluorescence Detection of Stx2 Activity

**Figure 2:**
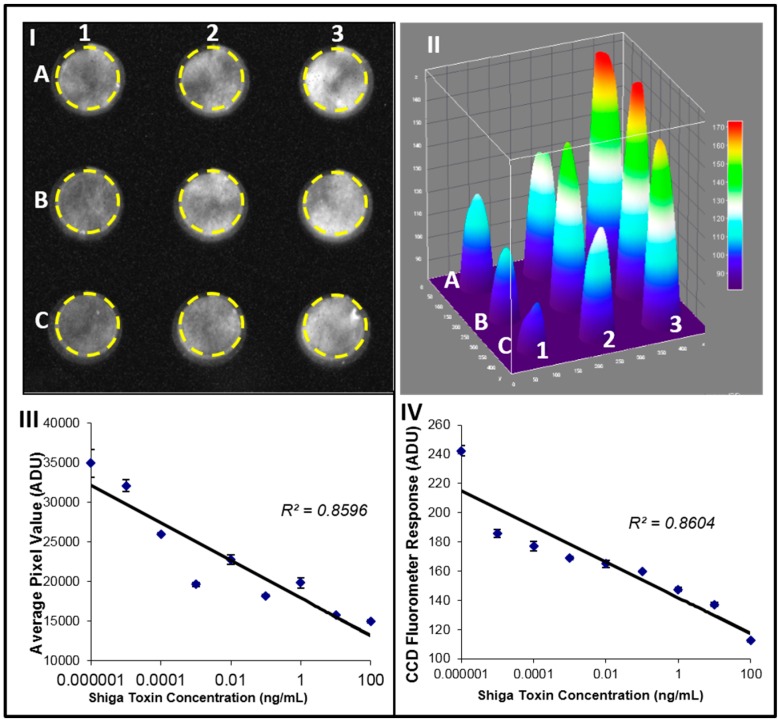
Fluorescence detection of Stx2 activity. Vero-GFP cell response to low concentrations of Shiga toxin. Vero-GFP cells were treated with various amounts of Shiga toxin. (**I**) signals were detected by the CCD operating in a still single frame mode. Toxin used: Control no toxin (3-A), 0.01 pg/mL (3-B), 0.1 pg/mL (3-C), 1 pg/mL (2-A), 10 pg/mL (2-B), 100 pg/mL (2-C), 1 ng/mL (1-A), 10 ng/mL (1-B), 100 ng/mL (1-C). (**II**) Corresponding ImageJ 3D image. (**III**) Average signal brightness (ADU) plotted against Shiga toxin concentration. (**IV**) Fluorimetric analysis of the same toxin concentrations.

One example to demonstrate the capability of such mobile optical detector is evaluation of the Shiga toxin (Stx2) activity [49]. The activity was measured as reduction of the cell green fluorescent protein (GFP) signal because of the toxin inhibition of the cell protein synthesis, GFP was measured with our fluorescence detector. Fluorescence-transduced cells with GFP construct were cultured for 24 h and treated with various amounts of Shiga toxin (100 ng/mL–0.01 pg/mL). In this work [49], the cells were imaged by the detector (Figure 2I) and the signal was quantified using ImageJ software (Figure 2II). In the control, which has no toxin, there is no signal inhibition (Figure 2II in well A3). It can be seen that the signal decreases in proportion to the level of toxin, up to the highest concentration level (100 ng/mL), as shown in well C3 of Figure 2II. In Figure 2III the average luminous intensity value was plotted against the Shiga toxin concentration. As a result of a high negative correlation between the fluorescence intensity signal and the concentration of the toxin, an increase in Stx2 concentration results in decreased GFP fluorescence intensity (*R*^2^ = 0.85, *p* value of 0.0037). The samples with the same toxin concentrations were also analyzed using a commercial fluorometer (Figure 2IV). Results indicate a very strong correlation between CCD and fluorometer measurements (*R^2^* = 0.86, *p* = 0.0030). The limit of detection (LOD) of the CCD camera was calculated as the mean pixel intensity value of the three control samples (cells with no toxin) minus three times the standard deviation of those samples. The LOD was determined to be 0.1 pg of Shiga toxin. This data suggests that a simple, low-cost CCD-based detector can be used as a fluorescent detector for cytotoxicity assays [49].

### 2.6. CCD-Based Detectors as Versatile Low-Cost Detectors for Food-Borne Toxins

A similar fluorescent detector configuration was used for botulinum neurotoxin A (BoNT-A) activity analysis [29,47,48]. The assay measures cleavage of a fluorophore-tagged peptide substrate specific for BoNT-A (SNAP-25) by the toxin light chain (LcA) with a sensitivity of 0.5 nM, which is the reported sensitivity of the SNAP-25 *in vitro* cleavage assays. Similar detectors were also used for food safety immunological assays to detect Staphylococcal Enterotoxin B (SEB) using enhanced chemiluminescence [129] with a LOD of 0.01 ng/mL, which is approximately 10 times more sensitive than traditional ELISA [121] for SEB detection utilizing colorimetric assays [120] or densitometry detection with a LOD of 0.5 ng/mL [148].

The simple optical configuration described above is sufficient for many fluorescence detection applications; however, there are many applications that require higher sensitivity. While cooled CCD cameras with low dark noise enable higher detection sensitivity [29,30,46,47,48,118,119,120,149,152,153], such devices are more expensive and may not be suitable for low-resource settings.

## 3. Improving the Sensitivity of Fluorescence Optical Detectors

As discussed above, there are several simple solutions to improve the sensitivity of low-cost optical devices. These include replacing the native lens of a webcam with a higher aperture lens, increasing the power of the excitation source in fluorescent detection, and longer exposure times to detect low signal. However, the longer exposure increases the image noise, so there is a need to develop optical approaches to improve sensitivity. Additional approaches to improve sensitivity of low-cost fluorescence detectors include: (1) the computational approach of image stacking; (2) the use of capillaries to improve fluorescence detection; (3) the use of lasers to enhance florescence excitation for flow cytometry; and (4) the use of streak imaging to capture the path of a single cell to detect rare cell events.

### 3.1. Computational Enhancement of the Sensitivity of Webcam-Based Detectors

The computational approach of image stacking has been used for sensitivity enhancement of digital images, which contain signals along with random noise and compression artifacts that reduce signal detection sensitivity as determined by the signal-to-noise ratio (SNR) [146,147,150]. In image stacking-based computational signal enhancement, many images of the same object are captured in video mode. Image stacking software then enables the average value of each pixel of each frame to be calculated to generate an “averaged” single image of all the frames. In the “average image,” the random noise of each frame will be averaged to a constant level after image stacking and the signal present in all frames will be a constant value in the averaged single image. In this way image stacking enables a reduction in the random noise and compression artifacts in video images by creating a “still average image” with improved SNR [150].

**Figure 3:**
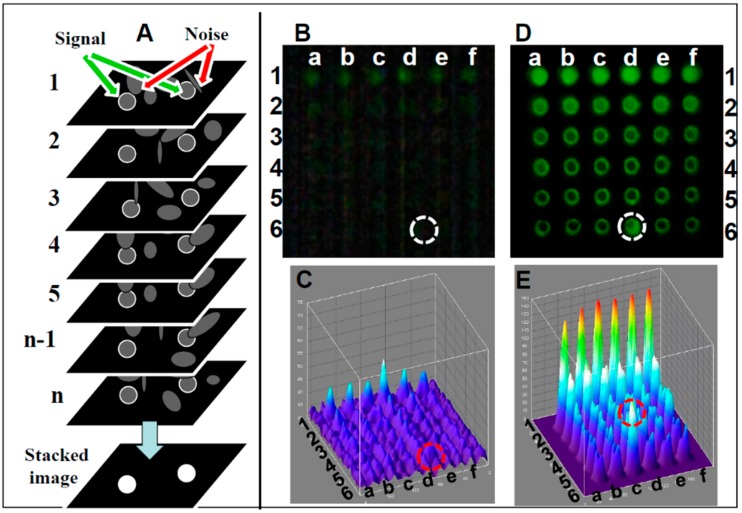
Enhancement of images captured from webcam video. (**A**) Schematic of image stacking for enhanced imaging. Webcam in video mode captures *n* individual frames; here *n* = 7. Each frame has an underlying signal of interest (white circles) and interfering noise. Standard deviation of background noise is greatly reduced if individual frames are averaged; the mean value can be subtracted from each pixel, resulting in enhanced SNR. A 36-well plate was loaded with six concentrations of fluorescein (rows 1–6), each replicated six times (columns a–f). The plate was illuminated by a blue LED equipped with a blue excitation filter and measured with a green emission filter. (**B**) Signals were detected by a CMOS webcam equipped with a f3.8 5-mm lens operating in a still single-frame mode. (**C**) Corresponding ImageJ 3D image. (**D**) The same plate, analyzed in video mode image, enhanced by image stacking. (**E**) Corresponding ImageJ 3D image. Fluorescein concentrations used: row 1: 500 μM, row 2: 250 μM, row 3: 125 μM, row 4: 60 μM, row 5: 30 μM, and row 6: control (water). Row 5 column d (circled) is a reference point used to orient the plate. SNR for all detectors was plotted against the various fluorescein concentrations.

- **Image stacking image analysis:** A schematic of the image stacking process is shown in Figure 3A. In video mode, the webcam captures *n* individual frames. Each frame captures pixels with a signal (marked with white circle) and pixels with random noise (marked with arrows). In high noise or low signal individual frames the signal and noise are indistinguishable, reducing the detection level. In the stacked image the random noise is subtracted, lowering random noise in the image while the signal remains constant, resulting in the increased SNR. A comparison between single frame and image stacking is described below.
- **Single frame Fluorescein detection:** A generic CMOS-based webcam used as a plate reader (Figure 1) equipped with the original 5 mm f3.8 lens was used for detecting Fluorescein (a common florescence dye used in many biological assays). In this experiment, samples in the range of 0–1 mg/mL were analyzed. In Figure 3B, an emission from a single frame of a 36-well plate (rows 1–6 in Figure 3A) with six replicas (columns a–f), where each row is loaded with six different concentrations of Fluorescein (0 mM (water) to 500 mM), is imaged using the webcam and analyzed with ImageJ software (NIH, Bethesda, MD, USA). Thus, it was possible to quantify the intensity of user-specified areas of the image. As shown in the still single frame of Figure 3B, the only signal detected in row 1 is the concentration of 500 μM, and there is no visible signal in the control (water, row #6) except in row 6, column d (marked with a circle), a reference point used to orient the plate. In the ImageJ 3D analysis (Figure 3C), the signal level for each well suggests that there is no strong signal except for the 500 μM (row 1) with an LOD (calculated based on the control (water in row 6) of 1000 μM.
- **Image stacking Fluorescein detection:** In video mode (30 frames per second), a stream of frames is captured for 10–15 s and saved as a compressed AVI file; this amounts to 300–450 frames. This file is then split into its constituent frames and averaged together through image stacking via ImageJ software [150]. Averaging serves to reduce the effects of random variation in the signal due to noise. Image stacking was used to improve CMOS sensitivity; the plate was detected by the CMOS webcam operating in a video mode enhanced by image stacking (Figure 3D) with the corresponding ImageJ image (Figure 3E), showing a very good signal with a LOD of 60 µM, an LOD similar to a conventional plate reader.

These results suggest that image stacking improves the sensitivity of inexpensive webcams, and that it may be practical to develop a low-cost fluorescence plate reader for around $100 with the sensitivity and capability to detect multiple analytes. Such a webcam-based plate reader can be used for telemedicine, enabling the transfer of data between distant locations to provide medical diagnostics from distant sites. In addition to fluorescence detection, such a plate reader can also be used for other optical modalities including absorbance, densitometry, and luminescence.

### 3.2. The Use of Low-Cost Lasers to Increase Light Excitation Combined with Streak Imaging to Improve Detection of Webcam-Based Portable Flow Cytometry

There are many clinical applications for rare cell detection, including circulating tumor cells (CTCs) for early cancer diagnosis and prognosis. In previous work [131,140,141] we have described a mobile flow cytometer that is suitable for the detection of such rare cells. The optical configuration of the flow cytometer is similar to the optical configuration of the webcam-based detector described above. To overcome the low sensitivity of the webcam, a commercially available low-cost blue laser without a lens was used for area-excitation of cells in a 2D flow cell that allows for high sample throughput at low velocities by using a wider flow field that overcomes the volume limitations associated with the hydrodynamics of flow focusing. The short exposure time (e.g., 1/20th of a second) affected the sensitivity of detection. Therefore, the solution was to replace the LED excitation source with a more powerful laser to increase the excitation energy. In addition, the use of streak imaging and image analysis tools enables improved detection of a webcam-based portable flow cytometer, reduces the size of the imaging files needed for application, reduces the time for analysis, and enhances the imaging capabilities of imaging sensors having high noise levels.

**Figure 4:**
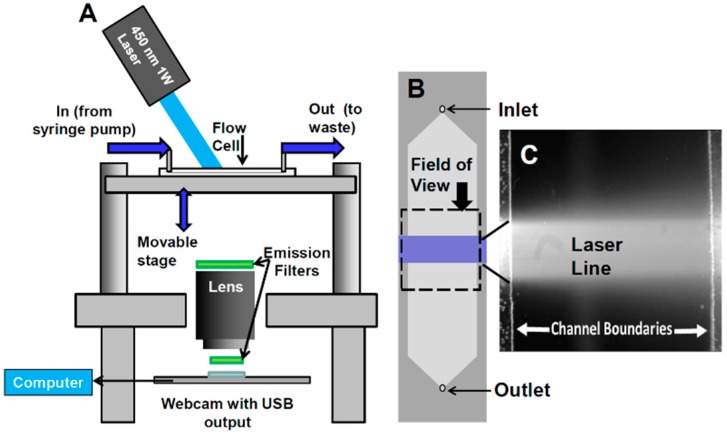
Schematic of webcam-based wide-field flow cytometer. (**A**) The flow cytometer consists of four modules: sensing element, excitation source, flow cell, and a stage to hold each module in alignment. The sensing element consists of the internal elements of a webcam, a 12-mm f/1.2 CCTV lens, two green emission filters, and a computer to collect and analyze data. The excitation source is a 450-nm 1W laser module. The sample handling module consists of a flow cell and a programmable syringe pump. (**B**) A schematic of the wide-field flow cell is shown with camera field of view and excitation laser line indicated, along with (**C**) an image from the camera showing the same features. The laser line is visible in this image due to autofluorescence of the glass flow cell.

**Figure 5:**
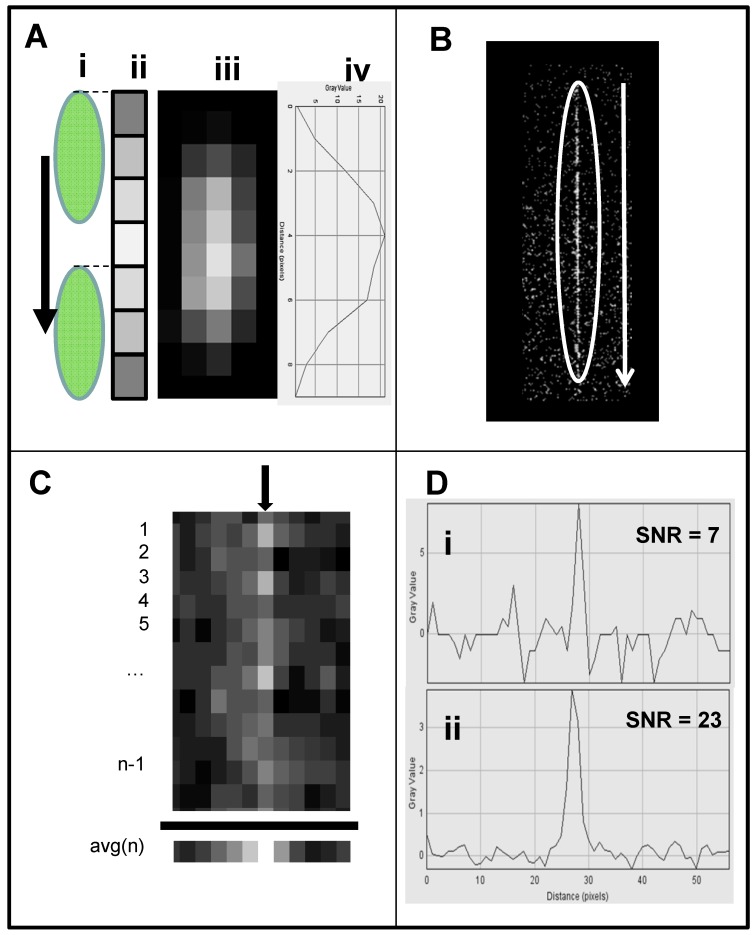
Streak mode imaging principles. (**A**) Schematic of a cell (i) traversing a number of pixels equal to its length plus one pixel, showing (ii) a maximum brightness achieved in the pixel at the image center. (iii) A cell image in streak mode is shown, along with (iv) a plot of its brightness along the center line of pixels. (**B**) A cell streak image (circled) with flow direction indicated. (**C**) Close-up of cell streak image showing individual pixels and background noise. In order to reduce noise, each column of pixels is averaged over the streak length *n* to produce a single averaged row of pixels, labeled avg(n). (**D**) A plot of pixel values before (i) and after (ii) averaging, showing ~three-fold improvement in SNR.

**Figure 6:**
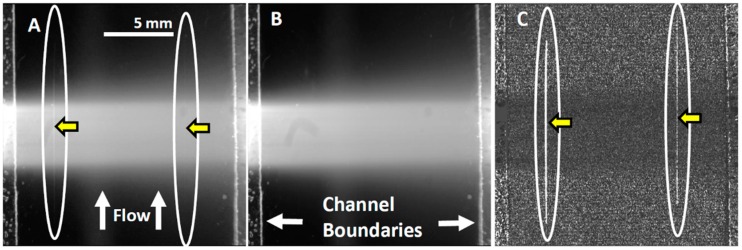
Streak imaging signal enhancement. Green channel video images of samples passing through the flow cell were enhanced to improve cell image visibility. (**A**) Single raw webcam image of human THP-1 monocytes stained with SYTO-9 dye; the cells are masked by the high fluorescence background and the positions of the fluorescent cell streak are circled and marked with arrows. The excitation laser line autofluorescence shown at the center. The average of all 720 video frames from one sample yields (**B**), a single frame containing only a background autofluorescent signal of the green video channel. The background (**B**) is subtracted from (**A**) to yield (**C**) a final image with improved cell streak visibility.

**Figure 7:**
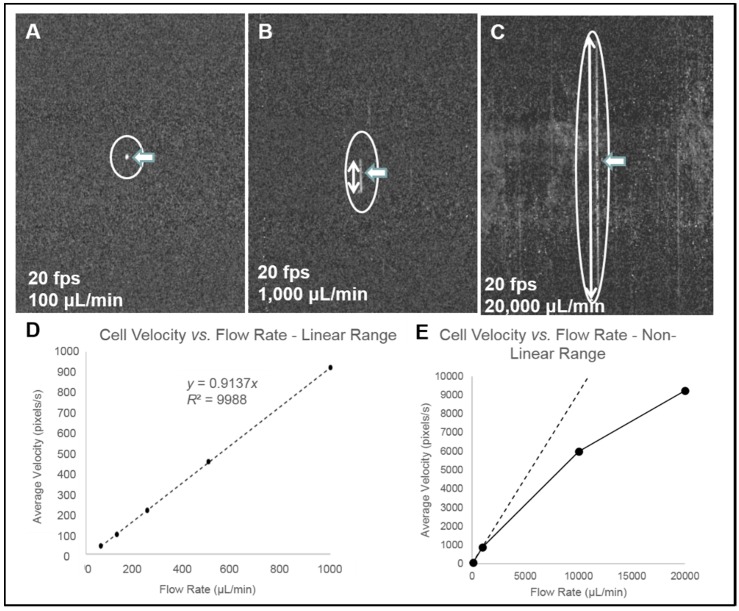
Streak image characteristics. (**A**–**C**) Three background-subtracted images of THP-1 monocytes (location and length indicated with arrows) captured at 20 fps are shown with varying flow rates. (**D**) The relationship between average cell velocity and flow rate in the linear range of flow cell operation, and (**E**) in the non-linear range of operation, with linear trend line plotted for comparison. Non-linearity in the relationship between flow rate and particle velocity is attributed to viscoelastic creep of the flow cell, resulting in increasing cross-sectional area at higher pressures.

- **Configuration of webcam-based mobile flow cytometer:** The mobile imaging flow cytometer (Figure 4A) is based on detector configuration of a webcam-based fluorescence plate reader (Figure 1). The optical system was adapted to close-up imaging (e.g. the use of extension tubes and focusing stage) and the LED illuminator (Figure 1A-6) was replaced with a laser. The new device (Figure 4) consists of four modules: (1) a webcam utilized as an imaging sensor; (2) a blue 450 nm 1W laser excitation source that enables high excitation energy and the detection of the cells using the low sensitivity detector; (3) a high throughput flow-cell (Figure 5B); and (4) a focusing stage for image focusing and alignment. The sensor includes the CMOS with the internal electronics of the webcam. The optical system includes a 12-mm f/1.2 CCTV lens, extension tube, and two green emission filters (no excitation filter was needed because of the narrow bandwidth for the laser illumination). The webcam was connected to a computer, which was used to power the webcam and to collect and analyze data. The fluid handling system includes a high throughput flow-cell (Figure 4B) and a programmable syringe pump.
- **Webcam-based flow cytometer wide-field imaging:** A high-throughput flow-cell (Figure 4B), which enables wide field rapid analysis and reduces the size of the imaging files used for analysis, was constructed in which (1) a glass or quartz microscope slide was used as a lower layer; (2) a middle layer laser was machined from 1.6 mm 3M 9770 double-sided adhesive transfer tape to define the geometry and depth of the fluid channel; and (3) a top layer comprising a glass or quartz microscope slide with two holes drilled for the inlet and outlet ports was aligned with the ends of the fluid channel layer. A wide flow channel (e.g., 20 mm) enables the sample flow rate to be increased and provides the capability to analyze the large sample volumes needed to detect rare cells. The wide cell enables imaging of cells moving a long distance in the flow cell, which maximizes the residence time of cells in the interrogation window of the field of view and maximizes the number of fluorescent cells imaged. The fluid volume of the interrogation window was maximized by the microscope-slide dimensions. The channel depth (~1.6 mm) kept the flow field within the depth of field of the lens being used (Pentax CCTV 12mm f/1.2, operated at approximately f/2.4 to reduce field curvature and improve depth of field). The lens was placed at a distance of approximately 20 mm from the webcam CMOS (using an extension tube), enabling the lens to focus at very close range on the entire detection field of the flow cell. To provide approximately uniform excitation across the width of the channel, the laser source was injected into the side of the flow cell (Figure 4C) at an angle that formed a linear band of excitation across the center of the field of view. For high sensitivity and high image quality, a Sony PlayStation^®^ Eye webcam was used as the imaging sensor.
- **Cell streak imaging cytometry:** Imaging of a large volume of moving cells was accomplished by increasing the flow cell volumetric rate up to to 20,000 μL/min. Images of the moving cells were obtained using “streak photography,” which allows imaging of moving objects at a low frame rate to be captured as short streaks in the final image (Figure 5B). Figure 6A shows schematically a fluorescently labeled cell traversing a number of pixels; the movement is captured by a CMOS detector on multiple pixels. The number of pixels corresponds to the cell distance, while the brightness of the pixels corresponds to the accumulation of the light emitted, with a maximum brightness achieved in the pixel at the image center (Figure 5A iii and iv). An actual image of such a cell is shown in Figure 5B. The direction and relative length of these streaks can be used to measure localized fluid motion. To further increase sensitivity, the signal-to-noise ratio of the images was also enhanced by combining three imaging methods: (1) CMOS color channel selection, (2) background subtraction, and (3) pixel binning [141]. Because the emission of the dye used (SYTO-9) is in the green range (498 nm), noise was reduced by using only the green pixels of the CMOS for the analysis and two green emission filters (on both sides of the lens, see Figure 4A). In order to reduce noise, as shown in Figure 5C, each column of pixels is averaged over the streak length *n* to produce a single averaged row of pixels, labeled avg(n). Figure 5D shows a plot of pixel values before (i) and after (ii) averaging, showing a three-fold improvement in SNR. The plot in (i) is for the row with the brightest pixel value quantitation, shown in Figure 5D.
- **Streak imaging signal enhancement:** To improve detection, only the green channel video images of samples passing through the flow cell were used to improve cell image visibility and reduce noise from the red and blue channels, which do not have significant green color signal. Figure 7A illustrates a single raw webcam image of human THP-1 monocytes stained with SYTO-9 florescence dye (with an excitation maximum at 483 nm and fluorescence emission maximum at 503 nm), showing a fluorescent cell streak (circled and marked with arrows) with the excitation laser line autofluorescence at the center. The average of all 720 video frames from one sample yields (B) a single frame containing only background autofluorescent signal of the green channel of video. This background (Figure 7B) is subtracted from each frame (Figure 5A) to yield an enhanced image (Figure 7C) with improved cell streak visibility.
- **Streak imaging cytometry detection of rare cells:** The relationship between volumetric sample flow rate, linear particle velocity, and the length of the streak in the wide field flow cell field of view is shown in Figure 7. In this experiment, CYTO-9 labeled THP-1 monocytes were injected at flow rates between 100 μL/min and 20 mL/min (Figure 7). The cells (marked with arrows) were captured at 20 fps (exposure time 1/20 s). The length of the streak is proportional to the flow rate. It was found that there were distinct linear (Figure 7D) ranges of operation. At higher flow rates (Figure 7E), non-linear cell velocity was measured, with a linear trend line plotted for comparison. Non-linearity in the relationship between flow rate and particle velocity is attributed to viscoelastic creep of the flow cell, resulting in increasing cross-sectional area at higher pressures.

To determine the counting efficiency of the described cell streak cytometer, the sample of human monocytes cells were counted and compared to counts obtained manually via fluorescent microscopy. Samples of cells at 1 cell/mL and 0.1 cell/mL concentrations were analyzed. For the target of 1 cell/mL, an average concentration of 0.91 cell/mL was measured by cytometry, with a standard error of 0.03 (C_95_ = 0.85–0.97). For the target of 0.1 cell/mL, an average concentration of 0.083 cell/mL was measured, with a standard error of 0.01 (C_95_ = 0.065–0.102). This data suggests that streak mode imaging enables detection of rare cells in the range of 1 cell per 10 mL. New software to automate cell counting of streak mode imaging cytometry is being developed (Ossandon *et al.*, unpublished results [154]); in preliminary results, for the target of 1 cell/mL, automated cell counting yields an average concentration of 0.87 cell/mL compared to 0.89 cell/mL for manual counting.

## 4. Cost Considerations for Global Health

While cost is an important consideration in low-resource settings for global health, it is very difficult to compare the price of commercial scientific instruments to a prototype system. In addition to the performance, the inherent capabilities, reproducibility, sensitivity, and reliability of the instruments will not be comparable, particularly for point-of-care diagnostics. However, in an effort to provide a very general estimate of the cost comparison, the price of a basic plate reader is ~$5500 (e.g., ACTGene AgileReader, Piscataway, NJ, USA), while the typical cost of the main components for a webcam-based fluorescence plate reader will be ~$200, as follows:

1. The webcam used as a detector ranges from ~$5 for a basic generic detector (from various suppliers, such as those found on eBay or Alibaba) to ~$10 for a Sony Playstation Eye webcam (eBay, San Jose, CA, USA).
2. Multi-wavelength LED White/Green/Blue/Red 48 LED SMD will cost ~$3 (eBay or Alibaba, Hangzhou, China).
3. Chroma filters will be one of the most expensive components at ~$70 (Chroma, Bellows Falls, VT, USA).
4. ImageJ Imaging software (NIH) for processing images is obtained as freeware.

Similarly, the cost of a basic plate flow cytometer (e.g., Accuri Cytometers, Franklin Lakes, NJ, USA) is ~$25,000. By contrast, the typical cost of the main components for a webcam-based flow cytometer will be ~$200:

1. Playstation Eye Webcam ~$10 (eBay)
2. Chroma filters ~$70 (Chroma)
3. Blue 450 nm 1W laser pointer ~$50 (eBay or Alibaba)
4. 12 mm f/1.2 CCTV lens ~$7 (eBay or Alibaba)
5. Peristaltic pump ~$6 (eBay or Alibaba) or syringe pump, Razel R-99 ~$160 (eBay)
6. ImageJ Imaging software (NIH) is free

## 5. Factors Contributing to Improving the Sensitivity of Mobile, Low-Cost Optical Devices for Fluorescent Detection

There are many factors that potentially contribute to improving the sensitivity of mobile, low-cost optical devices for fluorescent detection, making them acceptable for use in point-of-care diagnostics, albeit not all of them appropriate for global health applications. They include the following:

- *Webcams:* While lenses on smartphones are not interchangeable and require additional lens attachments to change the optics, resulting in degraded image quality, many webcams permit lenses to be easily changed (e.g., a *f*/1.2 lens can be used to maximize the amount of light transmitted to the sensor).
- *LEDs:* Increasing the power of the excitation source in fluorescent detection by increasing the intensity of the LED illumination (*i.e*., the use of more LEDs) increases the fluorescent signal.
- *Cameras:* Using cooled CCD/CMOS devices reduces thermal noise and improves SNR for more sensitive detection, but they are substantially more expensive than webcams.
- *Lasers:* The use of low-cost lasers equipped with line generator, or removing the laser lens, may increase light intensity and provide narrow wavelength illumination.
- *Exposure time:* For single frame imaging, some webcams allow for long exposure times (>1 s). Longer exposure can be used to detect faint optical signals; however, longer exposure times can also increase the thermal noise level in the images, requiring the active cooling found in more expensive cameras to control it.
- *Video imaging:* The use of video imaging mode combined with the image stacking computational approach results in an improved SNR.
- *Streak imaging:* The use of streak imaging with video mode enables the path of a cell to be captured over many pixels, which reduces the size of the imaging files needed for analysis. It also reduces the time for analysis, and enhances the imaging capabilities of imaging sensors having high noise levels.
- *Filters:* The quality of filters is very critical. Using high-quality, narrow band filters at the emission/excitation wavelengths reduces noise and improves detection.
- *Assays:* Fluorescence-based assays generating strong signals are ideal for low-sensitivity optical devices. For immunoassays, primary antibody immobilization can be enhanced by increasing the surface area for antibody binding through the use of nanoparticles, such as gold nanoparticles [121] or carbon nanotubes [118,119].

## 6. Conclusions

Optical technologies are important for biological analysis. While mobile consumer electronics devices are equipped with cameras that can be used for optical detection, the low sensitivity of these cameras limit their usefulness for biomedical applications. Examples have been provided of several low-cost approaches to improve the sensitivity of low-cost optical devices for biological analysis, including: (1) computational image stacking; (2) the use of capillaries to improve fluorescence detection; (3) lasers to enhance fluorescence excitation for flow cytometry; and (4) the use of streak imaging to capture the trajectory of a single cell enable imaging sensors with high noise levels to detect rare cell events. These examples demonstrate the potential for optically-based medical diagnostics that utilize readily available, low-cost consumer electronics capable of interconnectivity and telemedicine in point-of-care settings. Such imaging approaches substantially improve the detection level of consumer-grade optical devices, and can therefore be used to improve access to medical diagnostic technologies in low-resource settings for global health.
